# Acceptability of a COVID-19 Vaccine Among Healthcare Workers in the Kingdom of Saudi Arabia

**DOI:** 10.3389/fmed.2021.644300

**Published:** 2021-03-01

**Authors:** Ameerah M. N. Qattan, Noor Alshareef, Omar Alsharqi, Naseem Al Rahahleh, Gowokani Chijere Chirwa, Mohammed Khaled Al-Hanawi

**Affiliations:** ^1^Department of Health Services and Hospital Administration, Faculty of Economics and Administration, King Abdulaziz University, Jeddah, Saudi Arabia; ^2^Department of Finance, Faculty of Economics and Administration, King Abdulaziz University, Jeddah, Saudi Arabia; ^3^Economics Department, Chancellor College, University of Malawi, Zomba, Malawi

**Keywords:** acceptability, COVID-19, healthcare workers, hesitancy, Saudi Arabia, vaccine

## Abstract

**Objective:** This study aims to determine the acceptability of a COVID-19 vaccine among healthcare workers in Saudi Arabia and the factors affecting their intention to accept the vaccine.

**Methods:** The study used data from an online cross-sectional survey that was conducted in Saudi Arabia between 8 December 2020 and 14 December 2020. This study employed bivariate and multivariable regression analyses. The bivariate was used to describe and tabulate the frequency of all the variables, including the sociodemographic characteristics, the risk perception and the acceptance of the COVID-19 vaccination and a chi-squared test of independence was calculated. Multivariable logistic regression models were employed to examine and identify the factors associated with an intention to have the COVID-19 vaccination and the factors associated with its immediate acceptance.

**Results:** Of the total of 736 healthcare workers who began the online questionnaire, 673 completed it (a 91.44% completion rate). Among the study participants, 50.52% were willing to have the COVID-19 vaccine, of which 49.71% intended to have the vaccine as soon as it becomes available in the country, while 50.29% would delay until the vaccine's safety is confirmed. Being a male healthcare worker, perceiving a high risk of infection, and believing that the COVID-19 vaccine should be compulsory for all citizens and residents in the country increased the probability of intention to vaccinate against COVID-19 and the probability of accepting the COVID-19 vaccination as soon as possible.

**Conclusion:** This study calls for more health-related education among healthcare workers to alleviate any fears that might be associated with the COVID-19 vaccine.

## Introduction

The world is witnessing a major global humanitarian disaster due to the spread of the Coronavirus disease 2019 (COVID-19), which has affected all aspects of life across the planet. Countries around the world have implemented strict precautions and controls to contain the outbreak of COVID-19, which, among others, include social distancing and mandatory use of face coverings (1, 2). However, it is recognized that such preventive measures may neither be enough nor sufficient to halt the spread of COVID-19. Therefore, the vaccine's development and deployment is one of the most promising health intervention strategies to mitigate the spread of COVID-19 (3, 4).

COVID-19 vaccines are finally becoming available and many countries, including the Kingdom of Saudi Arabia (KSA), are already reserving supplies of the long-awaited vaccine. Following the Saudi Food and Drug Authority approval of the Pfizer-BioNTech COVID-19 vaccine, the country is set to introduce a phased vaccine rollout. Healthcare workers, the elderly, and patients with chronic and autoimmune diseases are scheduled to be early recipients of the vaccine (5). However, the success of any vaccination programme depends on high vaccine acceptance and uptake, and the main challenge that now lies ahead is building public confidence in an emergency-released vaccine. Without such confidence, vaccine hesitancy is immanent (6).

Vaccine hesitancy is defined as “the delay in acceptance or refusal of vaccination despite the availability of vaccination services,” and it is a global concern and a crucial factor in under-vaccination (7). Vaccine hesitancy presents a barrier to immunization program success and, in fact, has been identified by the World Health Organization (WHO) as one of the top 10 global health threats in 2019 (8). Despite the global effort to bring an end to the pandemic, anti-vaccination sentiments that spread misinformation on the dangers and consequences of vaccination cause hesitancy in immunization against preventable infectious diseases (9).

Healthcare workers play an important role in immunization program success and research has shown that their knowledge and attitudes in relation to vaccines determine their intentions for vaccine uptake and their recommendation of the vaccine (10, 11). There is a wealth of literature showing that healthcare workers can themselves be vaccine hesitant and their hesitancy levels can thus impact hesitancy and aversion to receiving the vaccine among the general public (12–14). Additionally, it has been reported that healthcare workers who have negative attitudes, are averted, or are hesitant about vaccinations share these unfavorable attitudes and tend to recommend vaccination to their patients infrequently (15).

Research studies assessing the uptake of seasonal and/or pandemic influenza vaccines among healthcare workers found that vaccine acceptance among this population is low. Various factors were found to underlie this behavior, which include low perceived benefits, low perceived risk of infection, fear of side effects and concerns surrounding safety and efficacy (16–19). Given the significant role of vaccinated healthcare workers on shaping the general population's decisions to vaccinate (20, 21), and as the availability of the vaccine does not necessarily translate into its adoption, this study thus aims to determine the COVID-19 vaccine's acceptability among healthcare workers in the KSA and to identify the factors affecting their intention to accept it. In this paper, healthcare workers are those who work in healthcare settings and deliver care and services to the sick and ailing either directly or indirectly such as physicians, dentists, nurses, pharmacists, and allied health professionals.

This study lands at a critical time for the Saudi health authorities as it is undertaken during the COVID-19 pandemic, specifically following the approval and before the arrival of the vaccine to the KSA. The results of this study are expected to provide insight into projected vaccine uptake and underlying drivers of vaccine-related decision making among healthcare workers. By understanding this, effective strategies can be developed to enhance COVID-19 vaccine uptake in the KSA, as well as in other countries in the Arabian Gulf. This study contributes to the limited literature on the demand (acceptability) of the novel COVID-19 vaccine in several ways. First, it assesses the demand for the vaccine across the healthcare workers who are not only at an increased risk of contracting and transmitting COVID-19 but whose acceptance of the vaccine is significant in preventing the transmission of the virus between medical personnel and patients. Second, this study represents one of the first findings on this matter in the KSA which is among the few countries that was able to successfully maintain a handle on the virus.

## Materials and Methods

### Study Design and Sample

This study used data from a cross-sectional survey that was conducted on the acceptability of a COVID-19 vaccine among the public and healthcare practitioners in the KSA from 8 December 2020 to 14 December 2020. The study recruited all participants from an online survey, via a self-reported questionnaire, using SurveyMonkey. Invitations to participate in the study were distributed to the respondents via Twitter and the WhatsApp communication platform. The participants were recruited using a simplified-snowball sampling technique where the invited participants were requested to pass the invitations to their WhatsApp contacts. The online approach is currently being used in order to avoid further physical contact as it might pose a risk of spreading the COVID-19 infection.

The target population was individuals aged 18 years or older and currently living in the KSA. Online informed consents were obtained from all participants before proceeding with the questions. The informed consent provided two options: “yes” for those who volunteered to participate in the study and “no” for those who did not wish to. Only those who selected the affirmative response were taken to the questionnaire page to complete the survey. The respondents were clearly informed about the study's aim and objectives and were also advised that they were free to withdraw from the study at any time, without giving a reason, and that all information and opinions provided would be anonymous and confidential.

### Measures

The self-reported questionnaire was designed and adapted by the authors based on similar studies and frameworks to assess vaccine acceptance for newly emerging infectious diseases (2, 7, 10, 22–24). The questionnaire was originally in English. M.K.A and N.A. translated the questions into Arabic, while A.M.N.Q and O.A. translated it back to English to ensure that the translation preserved the meaning captured by the original English version. The survey then used the Arabic text to administer the study.

The questionnaire consisted of 3 primary sections. The first section gathered information on the respondents' sociodemographic characteristics, including age, gender, marital status, education level, region in which they were currently residing, income level, and whether the healthcare practitioner was working on the front line in facing COVID-19. The second section collected information on the respondents' health status, vaccination history and perceived COVID-19 risk. The third section collected information on the acceptability of a COVID-19 vaccine.

### Statistical Analyses

The survey's primary outcome was the acceptance of the COVID-19 vaccination. In order to measure vaccination intention, the participants were asked about their willingness to be vaccinated. The respondents were provided with an informative statement that “scientists around the world are currently working on a vaccine that could prevent people from getting infected with COVID-19. It is hoped that the vaccine will become available in a few months.” The participants were then asked the following question “In the case that a COVID-19 vaccine becomes available in the next few months, with an effective rate of the COVID-19 vaccine between 90 and 95%, would you be willing to get the COVID-19 vaccine if it was provided free by the government?”. The respondents' options included “yes” or “no.” Respondents who stated “no,” that they are not willing to be vaccinated, were asked to indicate the main reason for their unwillingness to be vaccinated. Respondents who stated “yes,” and thereby showed a willingness to be vaccinated, were asked whether they would be willing to have the COVID-19 vaccine (to be vaccinated) as soon as possible when it became available or to delay vaccination until the vaccine safety was confirmed.

Some explanatory variables were collected. Respondents were asked about their sociodemographic characteristics, including their age, gender, marital status, the region in which they were residing, monthly income and whether they were working on the front line in facing COVID-19. The age variable was divided into five categories: 18–29 (the reference category), 30–39, 40–49, 50–59, and ≥60. Gender was coded as a dummy variable, with one for male and zero for female. Marital status was captured as binary, and a value of one was used for marriage and zero for otherwise (including single, widowed and divorced). Monthly income (Saudi Riyal, SR 1 = USD 0.27) was grouped into eight categories: <SR 3,000 (the reference category), SR 3,000 to <5,000, SR 5,000 to <7,000, SR 7,000 to <10,000, SR 10,000 to <15,000, SR 15,000 to <20,000, SR 20,000 to <30,000, and ≥SR 30,000. The healthcare workers status in relation to COVID-19 was also coded as one for those who are frontline healthcare workers and zero for otherwise. The region status covered all of the 13 administrative regions in the KSA, including Riyadh, Mekkah, Almadina Almonawra, Qaseem, Eastern Region, Aseer, Tabouk, Haiel, Northern Borders, Jazan, Najran, Albaha, and Aljouf, and was grouped into five categories, which are Central, West, East, North, and South.

Information was also collected on the healthcare worker respondents' health status, vaccination history and perceived COVID-19 risk. Respondents were asked whether they had a chronic illness that made them clinically vulnerable to serious illness from COVID-19 (yes, no), if they had been vaccinated for seasonal influenza (yes, no) and if they had ever refused a vaccine recommended by a physician because of doubts about it (yes, no).

The participants were also asked about psychological factors. The respondents were asked to what extent they thought COVID-19 poses a risk to people in Saudi Arabia, on a five-point Likert scale, from “no risk at all” to “major risk.” Additionally they were asked to what extent they are concerned about getting infected with COVID-19, on a five-point Likert scale, from “very low” to “very high.” They were also asked whether they have been infected with, or currently have, COVID-19 (yes, no), if any of their family members have been, or currently are, infected with COVID-19 (yes, no) and if any of their friends have been, or currently are, infected with COVID-19 (yes, no). The healthcare worker respondents were also asked whether they support that the COVID-19 vaccine should be compulsory for all citizens and residents inside Saudi Arabia or not.

This study employed bivariate and multivariable regression analyses. The bivariate analysis was done as cross-tabulation between all the variables and our dependent variable of interest using chi-squared tests. A multivariable logistic regression analysis was employed to examine and identify the variables associated with an intention to have the COVID-19 vaccination, with the odds ratio (OR), and a 95% confidence interval (CI) being calculated. Additionally, a multivariable logistic regression analysis was also performed to examine and identify factors associated with the vaccine demand group (immediate acceptance and delayed acceptance). All analyses were conducted using STATA 15.1 software (StataCorp LP, Texas, USA).

### Ethical Considerations

All procedures performed in this study involving human participants complied with the institutional and/or national research committee ethical standards and the 1964 Helsinki declaration and subsequent amendments or equivalent ethical standards. This research has been reviewed and given a favorable opinion by King Abdulaziz University. The study was designed and conducted in accordance with the ethical principles established by King Abdulaziz University and, therefore, ethical approval was obtained from the Biomedical Ethics Research Committee, Faculty of Medicine, King Abdulaziz University (Ref-628-20).

## Results

Of the total of 736 healthcare workers who began the online questionnaire, 673 completed it (a 91.44% completion rate). Among the 673 participants, 340 (50.52%) respondents were willing to have the COVID-19 vaccine if it was provided free by the government, while 333 (49.48%) were not willing to be vaccinated. Table 1 shows the frequency distribution of the intentions of COVID-19 vaccination acceptance by different the healthcare worker participants' characteristics and the factors that influence vaccination acceptance.

**Table 1:** Frequency distribution and chi-square analysis of intentions of COVID-19 vaccination acceptance.

**Variable**	**Willing to accept COVID-19 vaccination 340 (50.52%)**		**Not willing to accept COVID-19 vaccination 333 (49.48%)**		**Total**		***P*-value**
	**A**		**B**		**C**		**D**
**Age**							
18–29	80	(23.53%)	67	(20.12%)	147	(21.84%)	0.242
30–39	147	(43.24%)	158	(47.45%)	305	(45.32%)	
40–49	79	(23.24%)	62	(18.62%)	141	(20.95%)	
50–59	23	(6.76%)	33	(9.91%)	56	(8.32%)	
≥60	11	(3.24%)	13	(3.90%)	24	(3.57%)	
**Gender**							
Female	112	(32.94%)	156	(46.85%)	268	(39.82%)	0.000^***^
Male	228	(67.06%)	177	(53.15%)	405	(60.18%)	
**Marital status**							
Unmarried	106	(31.18%)	97	(29.13%)	203	(30.16%)	0.563
Married	234	(68.82%)	236	(70.87%)	470	(69.84%)	
**Location**							
Central	43	(12.65%)	52	(15.62%)	95	(14.12%)	0.000^***^
South	86	(25.29%)	37	(11.11%)	123	(18.28%)	
East	32	(9.41%)	42	(12.61%)	74	(11.00%)	
North	13	(3.82%)	9	(2.70%)	22	(3.27%)	
West	166	(48.82%)	193	(57.96%)	359	(53.34%)	
**Monthly income**							
< SR 3,000	43	(12.65%)	28	(8.41%)	71	(10.55%)	0.169
SR 3,000 to < SR 5,000	17	(5.00%)	9	(2.70%)	26	(3.86%)	
SR 5,000 to < SR 7,000	22	(6.47%)	15	(4.50%)	37	(5.50%)	
SR 7,000 to < SR 10,000	39	(11.47%)	49	(14.71%)	88	(13.08%)	
SR 10,000 to < SR 15,000	99	(29.12%)	97	(29.13%)	196	(29.12%)	
SR 15,000 to < SR 20,000	64	(18.82%)	68	(20.42%)	132	(19.61%)	
SR 20,000 to < SR 30,000	29	(8.53%)	28	(8.41%)	57	(8.47%)	
≥SR 30,000	27	(7.94%)	39	(11.71%)	66	(9.81%)	
**Frontline healthcare worker**							
No	157	(46.18%)	189	(56.76%)	346	(51.41%)	0.006^***^
Yes	183	(53.82%)	144	(43.24%)	327	(48.59%)	
**Having chronic conditions**							
No	270	(79.41%)	272	(81.68%)	542	(80.53%)	0.457
Yes	70	(20.59%)	61	(18.32%)	131	(19.47%)	
**Received flu vaccination in the past**							
No	79	(23.24%)	114	(34.23%)	193	(28.68%)	0.002^***^
Yes	261	(76.76%)	219	(65.77%)	480	(71.32%)	
**Refused vaccination in the past**							
No	312	(91.76%)	225	(67.57%)	537	(79.79%)	0.000^***^
Yes	28	(8.24%)	108	(32.43%)	136	(20.21%)	
**Infected with COVID-19**							
No	278	(81.76%)	281	(84.38%)	559	(83.06%)	0.365
Yes	62	(18.24%)	52	(15.62%)	114	(16.94%)	
**Family infected with COVID-19**							
No	201	(59.12%)	197	(59.16%)	398	(59.14%)	0.991
Yes	139	(40.88%)	136	(40.84%)	275	(40.86%)	
**Friends infected with COVID-19**							
No	27	(7.94%)	35	(10.51%)	62	(9.21%)	0.249
Yes	313	(92.06%)	298	(89.49%)	611	(90.79%)	
**Perceived risk of COVID-19 to people in Saudi Arabia**							
Minor risk or no risk	52	(15.29%)	70	(21.02%)	122	(18.13%)	0.010^**^
Moderate risk	118	(34.71%)	134	(40.24%)	252	(37.44%)	
Significant or major risk	170	(50.00%)	129	(38.74%)	299	(44.43%)	
**Concerned about getting infected with COVID-19**							
Low or very low	124	(36.47%)	158	(47.45%)	282	(41.90%)	0.000^***^
Fair	109	(32.06%)	116	(34.83%)	225	(33.43%)	
High or very high	107	(31.47%)	59	(17.72%)	166	(24.67%)	
**A COVID-19 vaccine should be compulsory for all citizens and residents inside Saudi Arabia**							
No	92	(27.06%)	314	(94.29%)	406	(60.33%)	0.000^***^
Yes	248	(72.94%)	19	(5.71%)	267	(39.67%)	

*** *p < 0.01*,

** *p < 0.05*.

Most of the healthcare worker participants were aged 30–49 (45.32%) and were male (60.18%). More than half of the respondents (60.18%) were married. About 29% of participants indicated that their income was in the range SR 10,000 to <SR 15,000, while only 10.55% of the participants were within the lower-income category of <SR 3,000.

327 respondents were frontline healthcare workers, thereby representing 48.59% of the sample. Four hundred eighty of the respondents (71.32%) received a flu vaccine in the past. About 17% had a history of being infected with COVID-19 and 20.21% had previously refused a vaccination recommended by a physician. Regarding the perceived risk of COVID-19 to people in Saudi Arabia, a majority (44.43%) perceived that it poses a significant or major risk to the people of Saudi Arabia, although many of the respondents (41.90%) thought that they had a minor or no risk of catching COVID-19. Suffice to say, a majority (60.33%) thought that the vaccine should not be compulsory [see column (C)].

As can be seen in Table 1, from column (D), it was found that gender, location, being a frontline healthcare worker, having received the flu vaccination in the past, having refused a vaccination recommended by a physician in the past, the perceived risk of COVID-19 to people in Saudi Arabia, the concern of being infected with COVID-19 and the participants' belief that the COVID-19 vaccine should be compulsory for all citizens and residents inside Saudi Arabia were all statistically significant.

It was also found that no significant association across age groups. Among those who showed a willingness to be vaccinated, more were male (67.06%), whereas, among those who said that they were not willing to be vaccinated, 46.85% were females. No significant association was observed across all income categories. Table 1 also lists additional results regarding the distribution of the other variables.

Table 2 shows the distribution of the vaccine demand group (the immediate acceptance group and vaccine delayed acceptance group) and the factors that influence vaccination–immediate or delayed–acceptance. Among 340 healthcare workers who were willing to be vaccinated, 169 (49.71%) respondents were willing to be vaccinated as soon as possible once the vaccine becomes available. On the other hand, 171 (50.29%) respondents would delay the vaccination until the vaccine's safety was confirmed.

**Table 2:** Frequency distribution and chi-square analysis of the vaccine demand group (immediate or delayed acceptance).

**Variable**	**Immediate acceptance 169 (49.71%)**		**Delayed acceptance 171 (50.29%)**		**Total**		***P*-value**
**Age**							
18–29	38	(22.49%)	42	(24.56%)	80	(23.53%)	0.899
30–39	73	(43.20%)	74	(43.27%)	147	(43.24%)	
40–49	40	(23.67%)	39	(22.81%)	79	(23.24%)	
50–59	11	(6.51%)	12	(7.02%)	23	(6.76%)	
≥60	7	(4.14%)	4	(2.34%)	11	(3.24%)	
**Gender**							
Female	47	(27.81%)	65	(38.01%)	112	(32.94%)	0.045^**^
Male	122	(72.19%)	106	(61.99%)	228	(67.06%)	
**Marital status**							
Unmarried	50	(29.59%)	56	(32.75%)	106	(31.18%)	0.529
Married	119	(70.41%)	115	(67.25%)	234	(68.82%)	
**Location**							
Central	18	(10.65%)	25	(14.62%)	43	(12.65%)	0.370
South	43	(25.44%)	43	(25.15%)	86	(25.29%)	
East	19	(11.24%)	13	(7.60%)	32	(9.41%)	
North	4	(2.37%)	9	(5.26%)	13	(3.82%)	
West	85	(50.30%)	81	(47.37%)	166	(48.82%)	
**Monthly income**							
< SR 3,000	17	(10.06%)	26	(15.20%)	43	(12.65%)	0.657
SR 3,000 to < SR 5,000	9	(5.33%)	8	(4.68%)	17	(5%)	
SR 5,000 to < SR 7,000	8	(4.73%)	14	(8.19%)	22	(6.47%)	
SR 7,000 to < SR 10,000	21	(12.43%)	18	(10.5%3)	39	(11.47%)	
SR 10,000 to < SR 15,000	54	(31.95%)	45	(26.32%)	99	(29.12%)	
SR 15,000 to < SR 20,000	31	(18.34%)	33	(19.30%)	64	(18.82%)	
SR 20,000 to < SR 30,000	16	(9.47%)	13	(7.60%)	29	(8.53%)	
≥SR 30,000	13	(7.69%)	14	(8.19%)	27	(7.94%)	
**Frontline healthcare worker**							
No	70	(41.42%)	87	(50.88%)	157	(46.18%)	0.080^*^
Yes	99	(58.58%)	84	(49.12%)	183	(53.82%)	
**Having chronic conditions**							
No	127	(75.15%)	143	(83.63%)	270	(79.41%)	0.0530^*^
Yes	42	(24.85%)	28	(16.37%)	70	(20.59%)	
**Received flu vaccination in the past**							
No	35	(20.71%)	44	(25.73%)	79	(23.24%)	0.2730
Yes	134	(79.29%)	127	(74.27%)	261	(76.76%)	
**Refused vaccination in the past**							
No	158	(93.49%)	154	(90.06%)	312	(91.76%)	0.250
Yes	11	(6.51%)	17	(9.94%)	28	(8.24%)	
**Infected with COVID-19**							
No	141	(83.43%)	137	(80.12%)	278	(81.76%)	0.429
Yes	28	(16.57%)	34	(19.88%)	62	(18.24%)	
**Family infected with COVID-19**							
No	106	(62.72%)	95	(55.56%)	201	(59.12%)	0.179
Yes	63	(37.28%)	76	(44.44%)	139	(40.88%)	
**Friends infected with COVID-19**							
No	14	(8.28%)	13	(7.60%)	27	(7.94%)	0.816
Yes	155	(91.72%)	158	(92.40%)	313	(92.06%)	
**Perceived risk of COVID-19 to people in Saudi Arabia**							
Minor risk or no risk	28	(16.57%)	24	(14.04%)	52	(15.29%)	0.217
Moderate risk	51	(30.18%)	67	(39.18%)	118	(34.71%)	
Significant or major risk	90	(53.25%)	80	(46.78%)	170	(50.00%)	
**Concerned about getting infected with COVID-19**							
Low or very low	52	(30.77%)	72	(42.11%)	124	(36.47%)	0.081^*^
Fair	57	(33.73%)	52	(30.41%)	109	(32.06%)	
High or very high	60	(35.50%)	47	(27.49%)	107	(31.47%)	
**A COVID-19 vaccine should be compulsory for all citizens and residents inside Saudi Arabia**							
No	26	(15.38%)	66	(38.60%)	92	(27.06%)	0.000^***^
Yes	143	(84.62%)	105	(61.40%)	248	(72.94%)	

*** *p < 0.01*,

** *p < 0.05*,

* *p < 0.1*.

As can be seen in Table 2, it was found that gender, being a frontline healthcare worker, being concerned about getting infected with COVID-19 and the participants' belief that the COVID-19 vaccine should be compulsory for all citizens and residents inside Saudi Arabia were all statistically significant. More males (72.19%) were willing to be vaccinated as soon as the vaccine becomes available than females. Table 2 also lists additional results regarding the distribution of the other variables.

Havin narrated the bivariate analysis, the next step is to present the multivariable logistic regression regarding the factors that are associated with the willingness to be vaccinated. These findings are reported in Table 3. For most age groups, there was no significant difference among the age groups, except for the 40–49 years old category, who were more likely to get vaccinated than those in the 18–29 years old category (OR: 2.226; 95% CI: 0.957–5.176). Furthermore, males were more likely to get vaccinated than females (OR: 1.609; 95% CI: 0.971–2.665). Healthcare workers living in the South were more likely to accept the vaccine (OR: 2.458; 95% CI: 1.047–5.775) compared to the people who indicated that they live in the Central region. In addition to the above, it is interesting to observe that no significant differences were observed across income quintiles, or being a frontline healthcare worker, having a chronic disease, and receiving a flu vaccine in the past.

**Table 3:** Logistic regression estimates of factors associated with acceptance of a COVID-19 vaccine.

**Variable**	**OR**	**95% CI**	***p*-value**
**Age**			
18–29	1		
30–39	0.969	0.486–1.931	0.929
40–49	2.226	0.957–5.176	0.063^*^
50–59	1.403	0.484–4.069	0.533
≥60	1.534	0.462–5.096	0.485
**Gender**			
Female	1		
Male	1.609	0.971–2.665	0.065^*^
**Marital status**			
Unmarried	1		
Married	0.802	0.418–1.539	0.506
**Location**			
Central	1		
South	2.458	1.047–5.775	0.039^**^
East	1.019	0.430–2.413	0.966
North	2.53	0.789–8.112	0.119
West	0.896	0.447–1.798	0.758
**Monthly income**			
< SR 3,000	1		
SR 3,000 to <5,000	1.763	0.453–6.864	0.413
SR 5,000 to <7,000	0.566	0.197–1.630	0.292
SR 7,000 to <10,000	0.5	0.173–1.444	0.200
SR 10,000 to <15,000	0.545	0.245–1.215	0.138
SR 15,000 to <20,000	0.669	0.274–1.631	0.377
SR 20,000 to <30,000	0.681	0.220–2.113	0.506
≥SR 30,000	0.799	0.269–2.372	0.686
**Frontline healthcare worker**			
No	1		
Yes	1.092	0.671–1.778	0.724
**Having chronic conditions**			
No	1		
Yes	0.658	0.342–1.268	0.212
**Received flu vaccination in the past**			
No	1		
Yes	1.328	0.804–2.193	0.268
**Refused vaccination in the past**			
No	1		
Yes	0.252	0.129–0.493	0.000^***^
**Infected with COVID-19**			
No	1		
Yes	1.841	0.893–3.795	0.098^*^
**Family infected with COVID-19**			
No	1		
Yes	1.19	0.72–1.97	0.497
**Friends infected with COVID-19**			
No	1		
Yes	1.402	0.633–3.103	0.405
**Perceived risk of COVID-19 to people in Saudi Arabia**			
Minor risk or no risk	1		
Moderate risk	1.521	0.803–2.883	0.198
Significant or major risk	1.86	0.955–3.624	0.068^*^
**Concerned about getting infected with COVID-19**			
Low or very low	1		
Fair	1.246	0.697–2.227	0.458
High or very high	2.091	1.068–4.092	0.031^**^
**A COVID-19 vaccine should be compulsory for all citizens and residents inside Saudi Arabia**			
No	1		
Yes	43.657	24.592–77.502	0.000^***^

*** *p < 0.01*,

** *p < 0.05*,

* *p < 0.1*;

*OR, odds ratio; CI, confidence interval*.

As can be seen in Table 3, healthcare workers who had ever refused a vaccine recommended by a physician because of doubts about it were less likely to be willing to be vaccinated (OR: 0.252; 95% CI: 0.129–0.493) compared with those who had never refused a vaccination. Another interesting result is concerning those who indicated that they were infected with COVID-19 in the past, as it showed that they were more likely to be vaccinated compared to those who had never been infected with COVID-19 (OR: 1.841; 95% CI: 0.893–3.795). The perceived risk of COVID-19 to people of Saudi Arabia and the concerns regarding catching COVID-19 were also associated with higher willingness to be vaccinated, as opposed to those who perceived the COVID-19 risk to people in Saudi Arabia as minor or no risk and those having low or very low concern of getting infected with COVID-19. Lastly, those who support that the vaccine for COVID-19 should be mandatory were more likely to express that they were willing to be vaccinated (OR: 43.654; 95% CI: 24.592–77.502).

Table 4 shows the analysis for the group that had shown willingness to be vaccinated only (*n* = 340). Multivariate logistic regression was performed between the immediate acceptance group (*n* = 169) and the delayed acceptance group (*n* = 171) to identify the factors that influence vaccination acceptance (immediate or delayed acceptance).

**Table 4:** Logistic regression estimates of factors associated with immediate or delayed acceptance of a COVID-19 vaccine.

**Variable**	**OR**	**95% CI**	***p*-value**
**Age**			
18–29	1		
30–39	0.677	0.321–1.425	0.304
40–49	0.718	0.297–1.736	0.462
50–59	0.616	0.219–1.736	0.360
≥60	1.425	0.227–8.949	0.705
**Gender**			
Female	1		
Male	1.706	0.986–2.952	0.056^*^
**Marital status**			
Unmarried	1		
Married	0.97	0.505–1.863	0.927
**Location**			
Central	1		
South	1.465	0.626–3.431	0.379
East	2.082	0.732–5.921	0.169
North	0.573	0.119–2.770	0.489
West	1.599	0.750–3.411	0.224
**Monthly income**			
< SR 3,000	1		
SR 3,000 to <5,000	1.974	0.565–6.895	0.286
SR 5,000 to <7,000	1.001	0.310–3.230	0.999
SR 7,000 to <10,000	2.454	0.796–7.562	0.118
SR 10,000 to <15,000	2.176	0.847–5.588	0.106
SR 15,000 to <20,000	1.775	0.631–4.997	0.277
SR 20,000 to <30,000	2.724	0.820–9.049	0.102
≥SR 30,000	2.547	0.696–9.312	0.158
**Frontline healthcare worker**			
No	1		
Yes	1.189	0.715–1.977	0.504
**Having chronic conditions**			
No	1		
Yes	1.375	0.725–2.607	0.330
**Received flu vaccination in the past**			
No	1		
Yes	1.09	0.603–1.970	0.776
**Refused vaccination in the past**			
No	1		
Yes	0.874	0.374–2.041	0.755
**Infected with COVID-19**			
No	1		
Yes	1.331	0.538–3.292	0.536
**Family infected with COVID-19**			
No	1		
Yes	0.849	0.512–1.408	0.525
**Friends infected with COVID-19**			
No	1		
Yes	1.111	0.456–2.710	0.816
**Perceived risk of COVID-19 to people in Saudi Arabia**			
Minor risk or no risk	1		
Moderate risk	0.59	0.291–1.193	0.142
Significant or major risk	0.763	0.391–1.491	0.429
**Concerned about getting infected with COVID-19**			
Low or very low	1		
Fair	1.72	0.825–3.586	0.148
High or very high	1.888	0.893–3.995	0.096^*^
**A COVID-19 vaccine should be compulsory for all citizens and residents inside Saudi Arabia**			
No	1		
Yes	3.666	2.034–6.608	0.00^***^

*** *p < 0.01*,

* *p < 0.1*;

*OR, odds ratio; CI, confidence interval*.

Among those who would accept vaccination, males (OR: 1.706; 95% CI: 0.986–2.952) were more likely to accept the COVID-19 vaccination as soon as possible once it becomes available when compared to females. Healthcare workers who perceived a high or very high risk of infection with COVID-19 (OR: 1.888; 95% CI: 0.893–3995) were more likely to accept COVID-19 vaccination as soon as possible once it becomes available than those who had a low or very low concern. Moreover, those who had the perception that a COVID-19 vaccine should be compulsory for all citizens and residents inside Saudi Arabia (OR: 3.666; 95% CI: 2.03–6.608) were also more likely to be willing to be vaccinated as soon as possible once it becomes available when compared to those who thought that the vaccine should not be mandatory.

Moving away from the bivariate and logistic regression analysis, it is also imperative to look into the reasons why people were not willing to get vaccinated and the findings pertaining to this aspect are shown in Table 5. Of the reasons put forward, many cited fears of adverse side effects from the vaccine (26.73%). The short duration of the clinical trials was also cited as a cause for concern (20.72%), which was followed by fear about the vaccine's safety, and efficacy (16.82%). The least among the reasons was that some thought that COVID-19 does not actually exist.

**Table 5:** Reasons for not accepting the COVID-19 vaccination.

	***N***	**%**
Fear of adverse side effects	89	26.73
Safety and efficacy concerns	56	16.82
The speed of making the vaccine	13	3.9
The short duration of clinical trials	69	20.72
Personal desire not to be vaccinated	30	9.01
I think the vaccine is a plot	32	9.61
I do not believe in the existence of COVID-19	2	0.6
I feel that masks and sanitisers are sufficient for protection	23	6.91
Other	19	5.71
Total	333	100

## Discussion

This study represents one of the first estimates of COVID-19 vaccination intention among healthcare workers in the KSA. Our findings can be used to guide future projections of vaccine uptake. Promoting the uptake of an emergency-released vaccine across a targeted population can pose significant challenges to public health authorities and in the context of the COVID-19 pandemic, failure to address such challenges could impede the country's unprecedented efforts in managing the pandemic. Thus, identifying the factors that can either be a facilitator or a barrier in influencing intentions to uptake or decline the COVID-19 vaccine is important.

The results reveal that almost half of the healthcare worker respondents in this study were unwilling to be vaccinated against COVID-19. 50.52% of the sample were willing to have the COVID-19 vaccine if it was provided free by the Saudi government, of which 49.71% were willing to be vaccinated as soon as the vaccine becomes available in the country, while 50.29% would delay vaccination until the vaccine's safety is confirmed. The vaccination acceptance rate was lower compared to earlier studies conducted in Saudi Arabia prior to the country's approval of the vaccine (25) or even before the vaccine was available (26).

Two reasons could explain this observed low rate. First, this study was conducted at the time when the Saudi government had just approved the COVID-19 vaccine. During that period, the dissemination of anti-vaccination misinformation on different social media platforms had intensified and this might have caused the creation of doubt about the novel vaccine. Second, the daily confirmed new COVID-19 cases in the country had started to decline at that time which could in turn resulted in alleviated worries among healthcare workers and contributed to weaker intentions to vaccinate.

Consistent with other previous findings from the United States of America (USA) (27), Australia (28), and Turkey (29) concerning the acceptance of the COVID-19 and influenza vaccinations, this study found that concerns regarding the vaccine's safety and efficacy and fear of adverse reactions were the most important predictors of vaccine refusal. Healthcare workers have also identified the expedited vaccine trials as a reason for lack of intent to vaccinate. Taken together these findings reaffirm results from previous studies of vaccine uptake during the influenza pandemic (30).

In the KSA, health authorities have highlighted that the Saudi Food and Drug Authority has stringent procedures in place to ensure the safety, effectiveness, and strengths of COVID-19 vaccine before permitting its use. They have also emphasized that approval came only after reviewing all scientific data that confirms the safety and efficacy of the vaccine, however uncertainties still exist (31). While there is a need to tailor effective outreach strategies aimed at addressing concerns related to vaccine safety and efficacy particularly among healthcare workers, the findings indicate that they need to be supplemented with building trust and ensuring transparency in the process of vaccine approval to achieve confidence and consequently improve vaccine acceptance.

In line with other studies (30, 32), the results of this study suggest an association between vaccine intention and healthcare workers' greater perceived risk of COVID-19 to themselves. It can thus be argued that the perceived risk of COVID-19 might remain even after being infected with the virus. The significant positive association between being previously infected with COVID-19 and vaccine intention found in this study supports this speculation.

Additionally, this study has found that vaccination intention was associated with a high-risk perception of COVID-19 to the country. The impact of the pandemic on the country's economic and social well-being had devastating consequences (33). Thus, it has been suggested that vaccination campaigns highlighting the pandemic's consequences on the overall country's well-being, including the social, economic and public cost of the disease, could be an effective strategy in encouraging vaccination (34). This strategy is especially important in Saudi Arabia, where coronavirus-related treatment has been offered to both residents and expatriates at no cost to curb the spread of the virus.

Given the global attention on COVID-19 vaccine nowadays, healthcare workers who believed that the COVID-19 vaccination should be mandatory were more willing to accept the vaccine. This could be stemming from the perception that the vaccine is the “seatbelt against the disease” and the potential solution in protecting oneself and others and achieving greater good at minimal cost (35).

In terms of vaccination history, vaccine intention was found to be correlated with previous acceptance of a certain type of vaccine. Earlier studies have identified habit (past vaccination behavior) as a strong determinant of future vaccination behavior (36). Previous study on influenza vaccine acceptance among healthcare workers in the KSA showed that the influenza vaccine uptake was low among healthcare workers, ranging from 3% in 2010 to 44.1% in 2015 (19). It has also been shown that the acceptance of a previous vaccination in Australia increased the intention to immunize, with participants who had accepted previous influenza vaccines being 5 times more likely to accept a pandemic vaccine (37).

There is some evidence suggesting that vaccination intention is likely to be higher than the actual vaccine uptake (38). In this study, almost 51% of those who were willing to be vaccinated intend to delay vaccination until the vaccine's safety is confirmed. Concerns regarding the safety of newly developed vaccines are well-documented (39–41). For example, 47% of Chinese people who showed an intention to accept the COVID-19 vaccination plan to delay immunization to see if there are associated side effects (23).

However, as the other half of the healthcare worker respondents who were willing to be vaccinated have the intention to vaccinate as soon as possible, it is important to identify the factors associated with immediate vaccination intention. Support for a mandatory vaccine was a significant predictor for immediate vaccination intention and healthcare workers who believe that vaccination should be mandatory were more likely to accept vaccination as soon as possible once the vaccine becomes available. Our results also confirmed risk perception's importance in accepting immediate vaccination, which concurs with the findings of other studies (23).

Furthermore, given that males are at high risk from COVID-19 (42), it was not unexpected that male healthcare workers were more willing to accept the COVID-19 vaccine compared to females healthcare workers. This finding is in line with several other studies (10, 23, 43). Additionally, we observed regional differences in COVID-19 vaccine acceptability. Healthcare workers residing in the Southern region of Saudi Arabia were more likely to report an intention to immunize against COVID-19 than residents of the Central region. While the reason behind this is unclear, it is important to note that the Southern region was among the worst-affected regions in the country and this could have played a role in promoting COVID-19 vaccination intention.

This study's strengths include the large sample size, participants from the 13 administrative regions in Saudi Arabia and the examination of a wide range of possible correlates. However, it is worthwhile looking at the possible limitations of the study and a key limitation is the study's cross-sectional design and lack of available data on non-respondents. Another limitation is that this study does not imply causality, given that it does not use causal identification methods. Finally, as the use of an online survey might impact the study's generalisability, it is worth noting that the sample of healthcare workers in this study is skewed toward the male gender (60.18% male, 30.82% female). According to the latest yearly statistical book by MOH in 2018 (44), the total male healthcare workers (including physicians, dentists, nurses, pharmacists, and allied health professionals) are 49.5% while the total female healthcare workers are 50.5%.

## Conclusion

This study provides early insight into the acceptability of the COVID-19 vaccine among healthcare workers in Saudi Arabia. Given that only half of the sample would be willing to be vaccinated, of which only half were willing to be vaccinated as soon as possible, it is worrying that the other half do not intend to be vaccinated, even though healthcare workers are expected to be more knowledgeable and aware of the benefits and risks of vaccination. There is an urgent need, therefore, to design effective and evidence-based strategies to promote the COVID-19 vaccine's uptake among healthcare workers. Healthcare workers are at great risk of contracting and spreading the disease and, unless wide-acceptance of the vaccine is achieved, the transmission of the virus would continue and recovery strategies would be hard to accomplish. Of particular importance is also the need for more health-related education among healthcare workers in order to alleviate any fears associated with the vaccine.

## Data Availability Statement

The datasets generated and analyzed during the current study are not publicly available due to privacy and confidentiality agreements as well as other restrictions, but are available from the corresponding author (Mohammed Khaled Al-Hanawi) on reasonable request.

## Author Contributions

All authors made substantial contributions to conception and design, acquisition of data, or analysis and interpretation of data, took part in drafting the article or revising it critically for important intellectual content, gave final approval of the version to be published, and agreed to be accountable for all aspects of the work.

## Conflict of Interest

The authors declare that the research was conducted in the absence of any commercial or financial relationships that could be construed as a potential conflict of interest.
